# Metabolic brain networks in aging and preclinical Alzheimer's disease

**DOI:** 10.1016/j.nicl.2017.12.037

**Published:** 2017-12-28

**Authors:** Katelyn L. Arnemann, Franziska Stöber, Sharada Narayan, Gil D. Rabinovici, William J. Jagust

**Affiliations:** a Helen Wills Neuroscience Institute, University of California Berkeley, Berkeley, CA, United States; b Leibniz Institute for Neurobiology, Magdeburg, Germany; c Clinic for Radiology and Nuclear Medicine, Otto-von-Guericke University, Magdeburg, Germany; d Memory and Aging Center, University of California San Francisco, San Francisco, CA, United States; e Division of Molecular Biophysics and Integrated Bioimaging, Lawrence Berkeley National Laboratory, Berkeley, CA, United States

## Abstract

Metabolic brain networks can provide insight into the network processes underlying progression from healthy aging to Alzheimer's disease. We explore the effect of two Alzheimer's disease risk factors, amyloid-β and ApoE ε4 genotype, on metabolic brain networks in cognitively normal older adults (N = 64, ages 69–89) compared to young adults (N = 17, ages 20–30) and patients with Alzheimer's disease (N = 22, ages 69–89). Subjects underwent MRI and PET imaging of metabolism (FDG) and amyloid-β (PIB). Normal older adults were divided into four subgroups based on amyloid-β and ApoE genotype. Metabolic brain networks were constructed cross-sectionally by computing pairwise correlations of metabolism across subjects within each group for 80 regions of interest. We found widespread elevated metabolic correlations and desegregation of metabolic brain networks in normal aging compared to youth and Alzheimer's disease, suggesting that normal aging leads to widespread loss of independent metabolic function across the brain. Amyloid-β and the combination of ApoE ε4 led to less extensive elevated metabolic correlations compared to other normal older adults, as well as a metabolic brain network more similar to youth and Alzheimer's disease. This could reflect early progression towards Alzheimer's disease in these individuals. Altered metabolic brain networks of older adults and those at the highest risk for progression to Alzheimer's disease open up novel lines of inquiry into the metabolic and network processes that underlie normal aging and Alzheimer's disease.

## Highlights

• Healthy aging and Alzheimer's disease had opposite effects on metabolic brain networks.
• Healthy aging led to widespread elevation of metabolic brain network correlations.
• Alzheimer's disease led to reduced metabolic brain network correlations.
• Independent metabolic function was lost in normal aging due to desegregation of ICNs.
• Together Aβ and ApoE ε4 altered the metabolic brain networks in old age.

## 1 Introduction

Distinct patterns of change in brain and cognitive functions dissociate the processes of healthy aging and Alzheimer's disease. Beyond the brain changes and gradual cognitive decline characteristic of normal aging (Park and Reuter-Lorenz, 2009), the hallmark of Alzheimer's disease is a stereotyped spatial pattern of neuritic plaques (amyloid-β or Aβ) and neurofibrillary tangles (tau), alongside loss of episodic memory and cognitive decline. Early identification of vulnerability to Alzheimer's disease - prior to the onset of clinical symptoms - is a central problem for the study of aging. PET imaging has revealed that some cognitively normal older adults harbor substantial Aβ (Sperling et al., 2011) and/or tau (Schöll et al., 2016) pathology, and are thought to be in a “preclinical” stage of Alzheimer's disease. Examining older adults with and without evident Alzheimer's disease pathology is necessary to dissociate brain changes of aging from the earliest stages of Alzheimer's disease.

Aging and Alzheimer's disease are associated with distinct changes in cerebral metabolism. Alzheimer's disease is associated with a characteristic pattern of cerebral hypometabolism in angular gyrus, posterior cingulate, precuneus, temporal, and parietal regions (de Leon et al., 1983, Minoshima et al., 1997). This pattern is distinct from that seen in normal aging, which is associated with hypometabolism in prefrontal, precentral, perisylvian, and anterior cingulate cortices (Chételat et al., 2013). The spatial pattern of hypometabolism is a reasonably sensitive biomarker for predicting future progression to Alzheimer's disease and can discriminate between normal aging, Alzheimer's disease, and other neurodegenerative diseases (Mosconi, 2005, Herholz et al., 2002). However, there is no consensus on changes in cerebral metabolism specific to preclinical Alzheimer's disease - while some studies detect hypometabolism (Drzezga et al., 2011, Lowe et al., 2014), other studies find hypermetabolism (Cohen et al., 2009, Oh et al., 2014), and others still find no differences in metabolism (Cohen et al., 2009, Altmann et al., 2015) associated with Aβ in normal aging and mild cognitive impairment (MCI).

Cerebral metabolism may not only be useful as a biomarker - it could play a causal role in the development of Alzheimer's disease pathology (Bero et al., 2011, Jagust and Mormino, 2011, Mosconi, 2013). Although the spatial pattern of atrophy and hypometabolism largely overlap in Alzheimer's disease, there is marked regional variability in their interrelationship, which suggests that hypometabolism may precede atrophy and possibly even pathology (Chételat et al., 2008). Sustaining high levels of metabolism may come at a cost (Bullmore and Sporns, 2012, Tomasi et al., 2013), the effects of which may be compounded across the lifetime (Jagust and Mormino, 2011) and induce vulnerability to Aβ deposition (Vlassenko et al., 2010, Oh et al., 2016). Highly metabolically active areas of the brain tend to be more highly connected (Tomasi et al., 2013) and exhibit a distinct pattern of gene expression (Goyal et al., 2014) compared to areas of the brain with lower metabolic demand. Further study of cerebral metabolism across the lifespan and prior to the onset of clinical symptoms is necessary to understand the role of metabolic processes in aging and the development of Alzheimer's disease.

However, these approaches are restricted to investigating univariate increases or decreases in metabolism between groups, whereas multivariate approaches may be more sensitive for investigating the relationship between Aβ and metabolism in the earliest stages of Alzheimer's disease. Researchers began looking at pairwise regional dependencies of glucose metabolism (Horwitz et al., 1984) near the advent of the use of [^18^F] fluorodeoxyglucose (FDG) to measure cerebral metabolic rate (Phelps et al., 1979). This approach has recently reemerged and grown in popularity, reconsidered in a network framework that allows for the application of the mathematical tools of graph theory (Bullmore and Sporns, 2009). By investigating pairwise dependencies across the brain, network approaches extract a shared pattern of covariation over time in the case of fMRI (Salvador et al., 2005) and electrophysiological methods (Bassett et al., 2006, Stam et al., 2007) or across subjects in the case of PET (Horwitz et al., 1986, Sepulcre et al., 2013), structural MRI (He et al., 2007), and gene expression (Richiardi et al., 2015). Studies of brain networks have revealed important insights into the phenomena of healthy aging and progression to Alzheimer's disease, including reduced connectivity affecting the main intrinsic brain networks (ICNs) in healthy aging (Sala-Llonch et al., 2015), profound reductions particularly to the default mode network in Alzheimer's disease (Dennis and Thompson, 2014), and accelerated desegregation of brain networks from healthy aging to Alzheimer's disease (Brier et al., 2014). However, potential reorganization of metabolic brain networks in aging and Alzheimer's disease progression remain poorly characterized.

Studies of metabolic brain networks, which measure co-variation in metabolism across individuals, complement univariate analyses of metabolism and other analyses of functional and structural brain networks. Metabolic brain networks are closely related to cortical thickness networks in that they estimate pairwise dependence of brain regions by examining correlations across individuals - just of metabolism measured by FDG PET, rather than cortical thickness measured by MRI (Alexander-Bloch et al., 2013). Early work on metabolic brain networks demonstrated age-related reductions of frontal-parietal metabolic correlations (Horwitz et al., 1986, Azari et al., 1992) and Alzheimer's-related reductions of metabolic correlation in frontal-parietal and homologous brain regions (Horwitz et al., 1987). More recent studies of MCI and Alzheimer's disease reported discrepant effects of ApoE genotype (Yao et al., 2015, Carbonell et al., 2014) and reduced metabolic correlation associated with Aβ in MCI (Carbonell et al., 2014). However, no studies have examined either (1) the joint effects of Alzheimer's disease risk factors (Aβ and ApoE ε4) in cognitively normal older adults, which have confounded studies of network function during resting state fMRI in normal aging (Brier et al., 2014) or (2) metabolic connectivity within- and between- canonical ICNs and graph theoretic properties of metabolic brain networks in cognitively normal aging. These gaps in knowledge obfuscate the link between metabolic brain networks and inquiries into aging, Alzheimer's disease progression, and brain network function in general.

It remains unclear whether Alzheimer's disease risk factors in cognitively normal older people will reflect a transitional stage between normal aging and Alzheimer's disease, if they will be indistinguishable from normal aging, or if they will demonstrate a unique profile of metabolic correlation. Using [^11^C] Pittsburgh compound B (PIB) -PET to divide cognitively normal subjects into groups based on Aβ (PIB- for low and PIB+ for high Aβ load) as well as ApoE genotype (ApoE ε4- and ApoE ε4+), we explore differences in properties of group metabolic brain networks using FDG-PET for young adults, subgroups of cognitively normal older adults, and patients with Alzheimer's disease.

## 2 Materials and methods

### 2.1 Participants

The study examined 17 young adults, 64 cognitively normal older adults, and 22 patients with Alzheimer's disease. All participants completed MR and PET imaging, as well as genetic testing for ApoE ε4 carrier status using previously published methods (Agosta et al., 2009). Because the ε4 polymorphism of the apolipoprotein E gene (ApoE) is a major genetic risk factor for Alzheimer's disease (Corder et al., 1993, Schellenberg, 1995), we stratified subjects based upon the presence of this allele as well as their Aβ status. Prior to participation all subjects provided informed consent in accordance with the Institutional Review Boards at UC Berkeley, UC San Francisco, and Lawrence Berkeley National Laboratory.

Young adults and cognitively normal older adults were recruited from the community via newspaper advertisements as part of the Berkeley Aging Cohort (BAC) at UC Berkeley. Subjects were required to live in the community independently, without any major medical, neurological, and psychiatric illnesses that could influence cognition; young adults were 18 to 30 years old and old adults were at least 60 years old. All subjects had scores on the Mini Mental State Examination ≥ 26 and performance on memory tests within 1.5 standard deviations of age-adjusted norms. The study included all eligible young adults who underwent both MR and FDG-PET imaging and were Aβ negative on PIB-PET scanning. From the population of cognitively normal older BAC participants meeting our criteria (N = 141), we formed four subpopulations based on PIB status (PIB- or PIB+, see Section 2.2.3) and ApoE ε4 carrier status (ApoE ε4- or ApoE ε4+), then identified the subgroup which included the fewest number of participants: those who were PIB- and ApoE ε4+ (N = 16). Three other subgroups (PIB- ApoE ε4-, PIB+ ApoE ε4-, and PIB+ ApoE ε4+) were then each formed by individually selecting 16 participants that best matched the demographic characteristics of participants in the PIB- ApoE ε4+ group based on age, gender, and years of education.

Alzheimer's disease patients were recruited at the University of California San Francisco Memory and Aging Center. Alzheimer's disease diagnosis was based on a comprehensive multi-disciplinary evaluation (Kramer et al., 2003); patients met criteria for probable Alzheimer's disease (McKhann et al., 2011), were Aβ positive on PIB-PET scanning, and were without any major comorbid medical, neurological, and psychiatric illnesses.

A single set of Alzheimer's disease and young controls were examined throughout the study; older control subjects were initially separated only by PIB status. For the remainder of analyses, the cognitively normal older adults were divided into four subgroups (N = 16) based on both PIB status and ApoE ε4 carrier status: Old PIB- ApoE ε4-, Old PIB- ApoE ε4+, Old PIB+ ApoE ε4-, and Old PIB+ ApoE ε4+ groups.

Table 1 shows the expected differences between groups in age, PIB index, and ApoE genotype based on group definitions. Two of the Alzheimer's disease participants were missing information - one did not undergo ApoE genotyping and another had an incomplete PIB scan and thus the PIB index could not be calculated but their Aβ positivity was confirmed through visual inspection by a clinician. We found no differences in gender and years of education between any of the groups, consistent with our sampling protocol.

**Table 1:** Group demographics.

	Young	Alzheimer's disease	Old PIB- ApoE4-	Old PIB- ApoE4+	Old PIB+ ApoE4-	Old PIB+ ApoE4+
# Subjects	17	22	16	16	16	16
Gender (female/male)	10/7	12/10	7/9	7/9	9/7	11/5
Age^a^	23.59 ± 2.79 (20 − 30)	74.82 ± 4.98 (69–89)	75.19 ± 3.68 (71–84)	74.81 ± 3.76 (71–83)	76.31 ± 3.23 (70–80)	75.23 ± 4.57 (69–89)
PIB Index^b^	0.98 ± 0.04 (0.92–1.05)	1.62 ± 0.25 (1.11–2.09)	1.01 ± 0.03 (0.96–1.06)	1.00 ± 0.08 (0.72–1.07)	1.26 ± 0.14 (1.09–1.54)	1.37 ± 0.24 (1.08–1.76)
# ApoE carriers^c^ (ε4+/ε4-)	6/11	14/7	0/16	16/0	0/16	16/0
Years of education	15.59 ± 1.68 (12 − 20)	16.27 ± 2.72 (12 − 22)	16.88 ± 2.23 (12–20)	16.88 ± 2.42 (12–20)	16.63 ± 1.36 (14–20)	16.75 ± 2.22 (12–20)
Scanner # ECAT/BIOGRAPH	13/4	15/7	11/5	8/7	8/8	7/9

a Young group < Old groups and Alzheimer's disease group.

b Young group and Old PIB- groups < Old PIB+ groups < Alzheimer's disease group.

c Young group < Old ApoE ε4+ groups; Old ApoE ε4- groups < Old ApoE ε4+ groups and Alzheimer's disease group.

### 2.2 Imaging acquisition and processing

#### 2.2.1 MRI

MR imaging of control subjects was performed at LBNL on a 1.5T Magnetom Avanto (Siemens Medical Systems) scanner using a 12 channel head coil. Structural scans were acquired axially using a high-resolution T1 MP-RAGE sequence (TR = 2110 ms; TE = 3.58 ms; TI = 1100 ms; flip angle = 15°; voxel dimension = 1.00 mm^3^; slice thickness = 1.00 mm with 50% gap).

MR imaging for Alzheimer's disease patients was performed at the Memory and Aging Center at UCSF on either a 1.5 T Siemens VISION System (N = 9) or 3 T Siemens Tim Trio (N = 13) scanner. Structural scans were acquired using high-resolution T1 MP-RAGE sequences, respectively acquired coronally with a quadracore head coil (TR = 10 ms; TE = 7 ms; TI = 300 ms; flip angle = 15°; voxel dimension = 1.00 mm^3^; slice thickness = 1.40 mm with no gap) and axially with a 12-channel head coil (TR = 2300 ms; TE = 2.98 ms; TI = 900 ms; flip angle = 9°; voxel dimension = 1.00 mm^3^).

The T1 MRI data underwent anatomical tissue segmentation using Freesurfer v5.3 (http://surfer.nmr.mgh.harvard.edu/) to produce 80 cortical and subcortical regions of interest (ROIs) in each subject's native space based on the Desikan-Killiany atlas. The segmentation was coregistered to PET using an inverted transformation of the affine mapping between the mean PET image and the skull-stripped brain in Anatomical Normalization Tools (ANTs; http://picsl.upenn.edu/software/ants/).

#### 2.2.2 PET

PIB- and FDG-PET imaging were performed at LBNL (ECAT EXACT HR or BIOGRAPH Truepoint 6 PET scanners in 3D acquisition mode), enabling in vivo measurements of Aβ and metabolism respectively. Imaging began with injection for 15-mCi of [^11^C] PIB, followed by 6 to 10-mCi of [^18^F] FDG at least 2-h later and included a 10-min transmission scan or an X-ray CT for attenuation correction. PIB-PET scanning began immediately upon injection, with dynamic acquisition frames obtained over 90-min (4 × 15 s, 8 × 30 s, 9 × 60 s, 2 × 180 s, 10 × 300 s, and 2 × 600 s). FDG-PET scanning began after 30-min of eyes-open quiet rest, with 6 × 5 min emission frames. Distribution volume ratio (DVR) images of PIB were produced by Logan graphical analysis with a cerebellar grey reference region. Standardized uptake value ratio (SUVR) images of FDG were produced with the pons as a reference region. Compared to other proposed FDG-PET reference regions, the pons has stable FDG tracer uptake across the aging and Alzheimer's disease spectrums (Minoshima et al., 1995). (For further details on PET acquisition and processing, see Wirth et al., 2013).

#### 2.2.3 PIB index

A PIB index was computed for each subject as the mean DVR across prefrontal, lateral temporal, parietal, and cingulate cortices, and was then used to separate the cognitively normal older subjects into Old PIB- (PIB index < 1.08) or Old PIB+ (PIB index ≥ 1.08) groups (Mormino et al., 2012). This threshold has previously been validated versus post-mortem Aβ burden (Villeneuve et al., 2015).

### 2.3 Metabolic brain network generation

Group metabolic brain networks were constructed for each group by computing Pearson's correlations of the FDG SUVR values across subjects between all pairs of ROIs. These correlations reflect relationships between brain regions across subjects, and are not based on canonical resting state networks but rather an approach used in graph theory in which the network reflects the interdependencies of all regions across the brain. FDG SUVR values were computed for each ROI by finding the mean SUVR value across all voxels within the ROI. This resulted in a fully weighted, symmetric 80 × 80 adjacency matrix for each group. The adjacency matrix was then converted to a fully weighted network, composed of 80 nodes (one for each ROI) and 3240 undirected weighted edges (one for each pairwise correlation between two ROIs, i.e. the values in the adjacency matrix).

### 2.4 Analysis

#### 2.4.1 Metabolic correlation strength

To summarize the metabolic correlation strength for each group, we computed the average correlation between all ROIs in the metabolic brain network. Metabolic correlation strength was computed on Fisher's Z-transformed correlation data, which was then inverse transformed back to correlation values with a possible range from − 1 to 1. We also computed metabolic correlation strength at the region level by averaging the strength of the correlations of each individual ROI with all other ROIs (Carbonell et al., 2014).

#### 2.4.2 Statistical testing

All descriptive statistics and statistical testing were performed on Fisher's Z-transformed data. We conducted ANOVA with a family-wise error rate of 0.05 to test for group differences in analyses of demographic data and metabolic brain networks, followed by Tukey's HSD post-hoc test to examine pairwise differences between groups. We conducted chi-squared tests to test for group differences of dichotomous demographic data followed by pairwise differences between groups, adjusting the p-value using Bonferroni correction.

#### 2.4.3 Permutation testing of regional differences in correlation strength

The statistical significance of differences in mean regional correlation strength between groups was estimated using permutation testing. We pooled subjects in the two groups under comparison, and then randomly assigned N subjects to the first group and the remaining subjects to the second group, extracting their FDG SUVR data to generate group metabolic brain networks. We then computed differences in mean regional correlation strength between the groups. Differences were computed on Fisher's Z-transformed data before being transformed back into correlations with a possible range from − 1 to 1. We repeated this procedure 100,000 times, with the results used to estimate a 95% confidence interval of group differences. If the empirical value of the difference between groups lay outside the 95% confidence interval of differences produced by this random assignment procedure, then we rejected the null hypothesis and the empirical difference between the groups was deemed significant.

#### 2.4.4 Control for spatial proximity based on anatomical distance

We computed the Chebyshev distance (the number of grey matter voxels that must be traversed to connect 2 points) between the centers of mass for each pair of ROIs using the Freesurfer average brain parcellation in MNI152 space. We computed the center of mass for each ROI using Chebyshev distance in a similar manner, by finding the voxel within each ROI that minimized the number of grey matter voxels that must be traversed to connect the voxel and all other voxels within the ROI. We deemed connections long-distance if the Chebyshev distance was above the median of all pairwise distances. We then employed statistical testing, as described in Section 2.4.2 to compare group differences in metabolic correlation strength for only long-distance connections.

#### 2.4.5 Intrinsic connectivity network analysis

Using the functional atlas proposed by Shirer et al. (2012), composed of 84 ROIs associated with 14 intrinsic connectivity networks (ICNs), we sought to compare features of metabolic correlations within- and between-ICNs derived from resting-state fMRI. The ICNs are composed of ROIs that functionally coordinate in the absence of evoked activity, i.e. during a “resting” scenario similar to that in which the participants engaged during the FDG-PET scan. To examine the metabolic correlations within- and between-ICNs, we used the approach described in Section 2.3 to generate metabolic brain networks from the 84 ROIs in the functional atlas. We then computed the metabolic correlation strength within each ICN and between each pair of ICNs. We computed within-ICN correlation strength for a given ICN by calculating the average correlation strength of the connections between all ROIs within an ICN. Given two ICNs, we computed between-ICN correlation strength by calculating the average correlation strength of the connections between all ROIs in one ICN and all ROIs in the other ICN. As described in Section 2.4.1, metabolic correlation strengths were calculated by averaging Fisher's Z-transformed data, before inverse-transforming the data to correlation values between with a possible range of − 1 to 1.

## 3 Results

### 3.1 Group differences in metabolic brain networks when normal older groups dichotomized by Aβ alone

#### 3.1.1 Qualitative differences in metabolic brain network correlation matrices

**Fig. 1:**
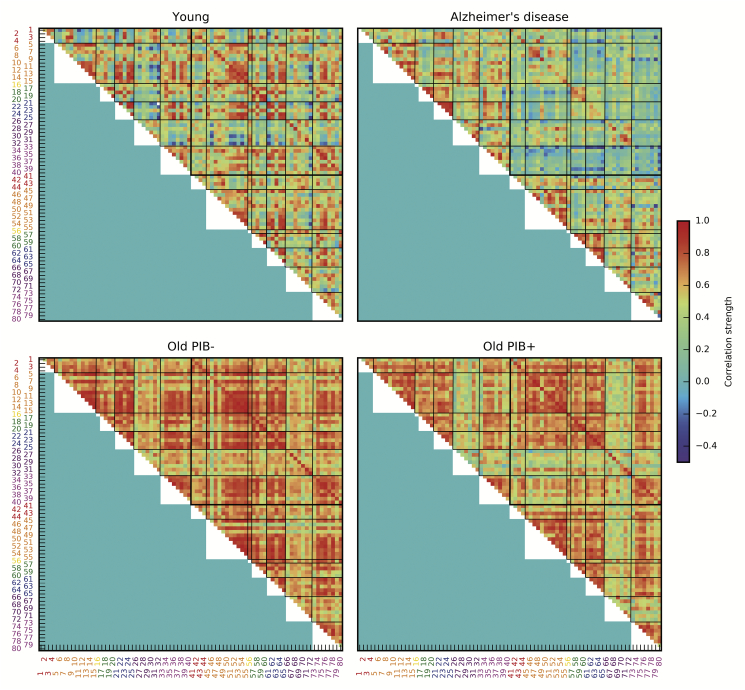
Group metabolic adjacency matrices. Adjacency matrices are composed of pairwise correlation strength between all ROIs shown for young, Alzheimer's disease, Old PIB-, and Old PIB+ groups. Regions of interest are numbered with label color corresponding to lobe membership: red = cingulate, orange = frontal, yellow = insula, green = occipital, blue = parietal, purple = subcortical, magenta = temporal. (For interpretation of the references to color in this figure legend, the reader is referred to the web version of this article.)

Adjacency matrices of the metabolic brain networks are shown in Fig. 1, revealing qualitative differences in the pattern of metabolic correlation strengths in the young, Alzheimer's disease, Old PIB-, and Old PIB+ groups, prior to splitting the cognitively normal older adults into four subgroups. The Young group exhibits the most heterogeneous pattern of correlations, with the strength of the correlations ranging from moderate negative correlations to strong positive correlations, and a combination of strong local (i.e. within-lobe) and distant (i.e. between-lobe) associations. Although the Alzheimer's disease group also exhibits a relatively heterogeneous pattern of correlation strengths, a notable characteristic of the Alzheimer's disease group is reduction in correlation strength of homologous brain regions and between hemispheres (respectively the diagonal and off-diagonal of the upper right quadrant of the adjacency matrix in Fig. 1). Both the Old PIB- and Old PIB+ groups exhibit a homogenous increase in correlation strength across cortical (and to a lesser extent subcortical) ROIs relative to young adults and patients with Alzheimer's disease, with smaller increases in correlation strength in the Old PIB+ group.

#### 3.1.2 Mean metabolic correlation strength

The ANOVA examining group differences in mean metabolic correlation strength revealed a significant difference between the groups (F = 1159.01, p ≪ 1.00e^− 10^, df = 12,636). The highest mean correlation strengths were seen in the Old PIB- group, followed by the Old PIB+, the young, and the Alzheimer's disease groups (Tukey's HSD post-hoc test FWE = 0.05).

### 3.2 Group differences in metabolic brain networks when normal older groups dichotomized by both Aβ and ApoE 4 genotype

#### 3.2.1 Qualitative differences in metabolic brain network correlation matrices

**Fig. 2:**
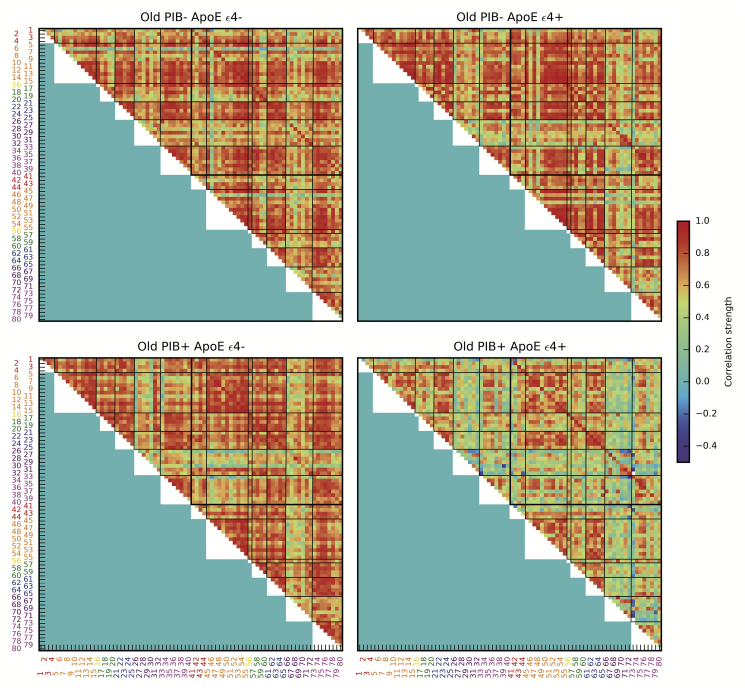
Older subgroup metabolic adjacency matrices based on Aβ and ApoE genotype. Adjacency matrices are composed of pairwise correlation strength between all ROIs shown for Old PIB- ApoE ε4-, Old PIB- ApoE ε4+, PIB+ ApoE ε4-, and Old PIB+ ApoE ε4+ groups. Regions of interest are numbered with label color corresponding to lobe membership: red = cingulate, orange = frontal, yellow = insula, green = occipital, blue = parietal, purple = subcortical, magenta = temporal.(For interpretation of the references to color in this figure legend, the reader is referred to the web version of this article.)

Adjacency matrices of the metabolic brain networks for older subjects defined by Aβ and ApoE genotype are shown in Fig. 2. The Old PIB- ApoE ε4-, Old PIB- ApoE ε4+, and Old PIB+ ApoE ε4- groups all exhibit a similar homogeneous pattern of increased correlation strength between most cortical ROIs compared to the young and Alzheimer's disease groups in Fig. 1. This indicates that across subjects, the metabolic relationships between region-pairs are relatively consistent. However, the Old PIB+ ApoE ε4+ group exhibits a distinct, more heterogeneous pattern of correlation than other cognitively normal older adult subgroups, indicating that across all subjects, metabolism in one region inconsistently predicts metabolism in another region compared to other cognitively normal older adults. While the overall pattern of correlation strength may be dampened for the Old PIB+ ApoE ε4+ group relative to other subgroups of cognitively normal older adults, the reduced correlation strength of the Old PIB+ ApoE ε4+ group is particularly prominent in cingulate and temporal lobe ROIs.

#### 3.2.2 Mean metabolic correlation strength

**Fig. 3:**
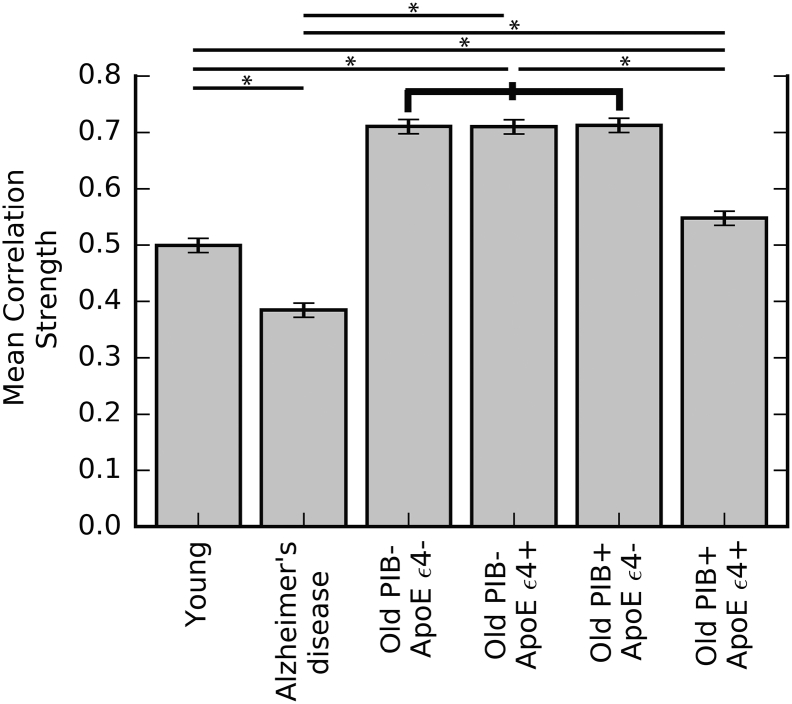
Group differences in metabolic correlation strength. Error bars show the simultaneous confidence intervals from Tukey's HSD post-hoc test. * p ≤ 0.05.

The ANOVA examining group differences in mean metabolic correlation strength revealed a significant difference between the groups (F = 1151.30, p ≪ 1.00e^− 10^, df = 18,954). The highest mean correlation strengths were seen in the Old PIB- ApoE ε4-, Old PIB- ApoE ε4+, and PIB+ ApoE ε4- groups, which were not significantly different from one another, while all other groups differed (Tukey's HSD post-hoc test FWE = 0.05) (Fig. 3). The Old PIB+ ApoE ε4+ group was intermediate in correlation strength between the young subjects and the other old subjects.

#### 3.2.3 Mean regional metabolic correlation strength

**Fig. 4:**
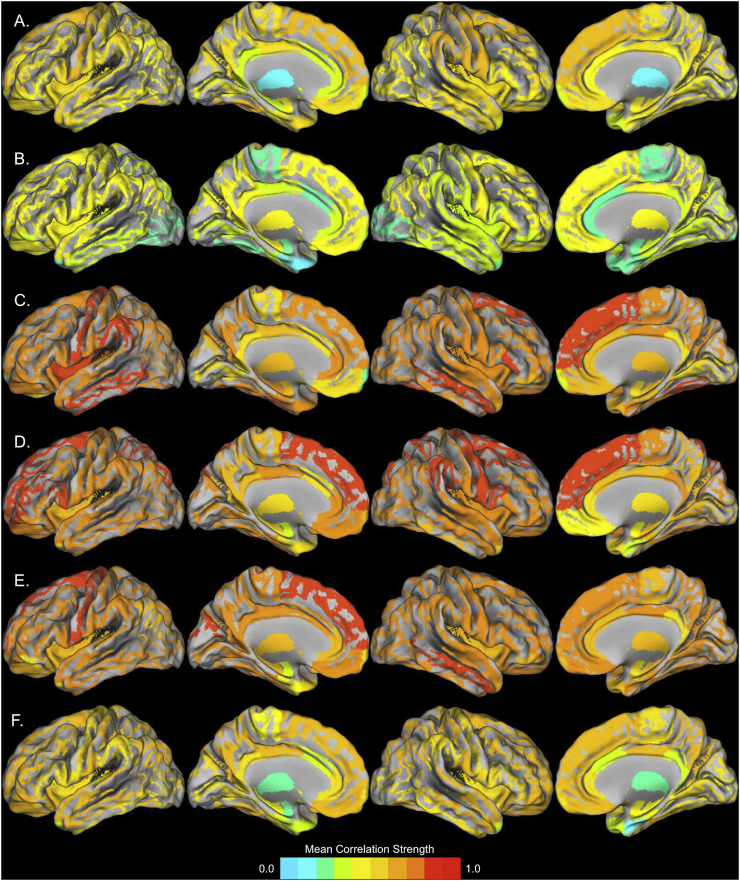
Mean regional metabolic correlation strength for each group. A. Young B. Alzheimer's disease C. PIB- ApoE ε4- D. PIB- ApoE ε4+ E. PIB+ ApoE ε4- F. PIB+ ApoE ε4+. Regions with high metabolic correlation strength are metabolic brain networks “hubs”.

We examined the mean regional metabolic correlation strengths of each group's metabolic brain network by computing the average correlation strength of each ROI with all other ROIs. All groups exhibited relatively high correlation strengths in frontal, parietal, and lateral and superior temporal ROIs and relatively low strengths observed in medial temporal lobe, temporal pole, cingulate, and subcortical ROIs (Fig. 4). Young adults and Alzheimer's patients show distinct patterns of regional metabolic correlation strength. Similar patterns of relative correlation strength emerged across the subgroups of older adults, although the PIB+ ApoE ε4+ subgroup appeared to be in an intermediary stage between normal aging and Alzheimer's disease.

#### 3.2.4 Group differences in mean regional metabolic correlation strength

**Fig. 5:**
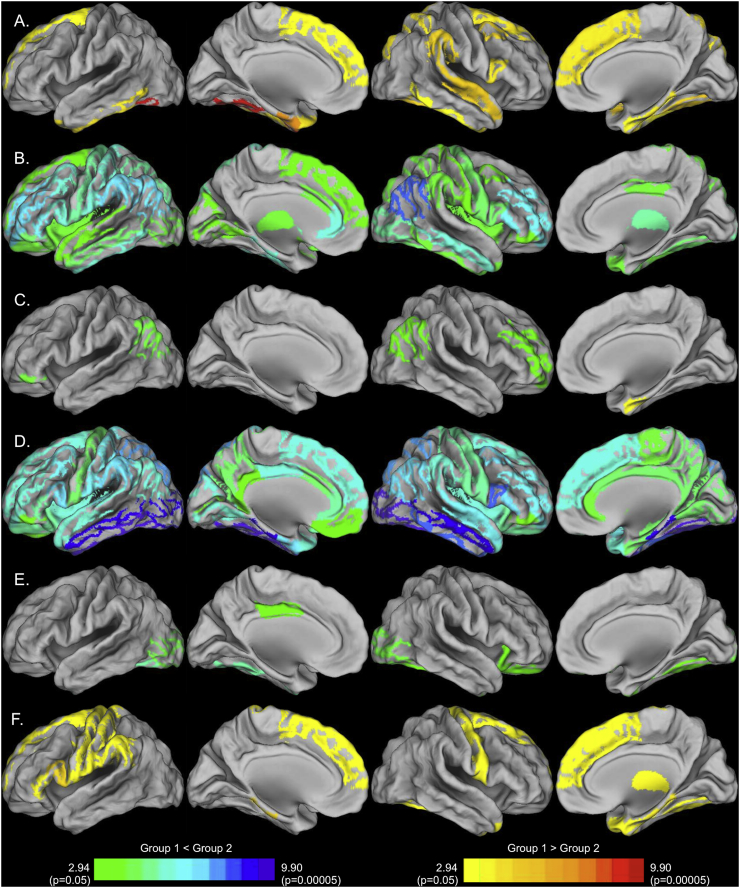
Group differences in regional metabolic correlation strength. A. Young > Alzheimer's disease (warm) and Young < Alzheimer's disease (cool) B. Young > Other Old (warm) and Young < Other Old (cool) C. Young > Old PIB+ ApoE ε4+ (warm) and Young < Old PIB+ ApoE ε4+ (cool) D. Alzheimer's disease > Other Old (warm) and Alzheimer's disease < Other Old (cool) E. Alzheimer's disease > Old PIB+ ApoE ε4+ (warm) and Alzheimer's disease < Old PIB+ ApoE ε4+ (cool) F. Other Old > Old PIB+ ApoE ε4+ (warm) and Other Old < Old PIB+ ApoE ε4 + (cool). The significance of the difference between groups is indicated by the region's color, in terms of the logit of the uncorrected p-value obtained via permutation testing.(For interpretation of the references to color in this figure legend, the reader is referred to the web version of this article.)

We examined the topography of group differences in mean regional metabolic correlation strength using permutation testing (Fig. 5). Permutation testing revealed minimal differences between the Old PIB- ApoE ε4-, Old PIB- ApoE ε4+, and Old PIB+ ApoE ε4- groups. For this reason, we combined the results from these groups to simplify the analysis. This resulted in a comparison between the young, Alzheimer's disease, Old PIB+ ApoE ε4+, and all other cognitively normal older subjects (i.e. “Other Old group”, N = 48).

The Young group differed profoundly from the Other Old group (Fig. 5B) showing widespread reductions in correlation strength, but exhibited less extensive differences with the Alzheimer's disease group (Fig. 5A) and the Old PIB+ ApoE ε4+ group (Fig. 5 C). Of interest, the Old PIB+ ApoE ε4+ group exhibited significant reductions relative to the Young group in right entorhinal cortex, and increases relative to the Young group in ROIs in the left pars orbitalis, right rostral middle frontal, and bilateral inferior parietal cortex. The Old PIB+ ApoE ε4+ showed relatively few differences with the Alzheimer's disease group (Fig. 5E), but exhibited moderate differences with the Other Old group (Fig. 5F). A notable commonality between the Old PIB+ ApoE ε4+ and the Alzheimer's disease groups is the low metabolic correlation strength in ROIs in the entorhinal cortex and medial temporal lobe compared to young and other old subjects.

### 3.3 Control for spatial proximity

Because partial volume effects of age- and disease-related atrophy may artificially inflate the metabolic correlation between spatially proximal regions (i.e. regions with short anatomical distance), we: (1) examined the relationship between the spatial proximity and correlation strength for all pairs of regions and (2) performed statistical testing for group differences in mean metabolic correlation strength on only long-distance connections that eliminate any shared effect of spatial proximity on correlation strength.

The Alzheimer's disease group demonstrated substantial decay in correlation strength with increasing anatomical distance (R = − 0.52, p ≪ 1.00e^− 10^), and a small but significant negative relationship was also found in the Young (R = − 0.07, p = 3.45e^− 5^), Old PIB- ApoE ε4- (R = − 0.09, p = 4.38e^− 7^), and Old PIB+ ApoE ε4- (R = − 0.11, p = 8.51e^− 10^) groups. No relationship was found in the Old PIB+ ApoE ε4+ group and a small but significant positive relationship was found in the Old PIB+ ApoE ε4- group (R = 0.05, p = 0.007). Group differences in mean correlation strength persisted even after examining only long-distance connections (F = 927.24, p ≪ 1.00e^− 10^, df = 9348), where Tukey's HSD post-hoc test revealed significant differences between all groups except the Old PIB- ApoE ε4- with the Old PIB- ApoE ε4+ and Old PIB+ ApoE ε4- groups. The overall pattern of relative metabolic correlation strengths was identical to those reported in Section 3.2.2.

### 3.4 Intrinsic connectivity network connectivity

**Fig. 6:**
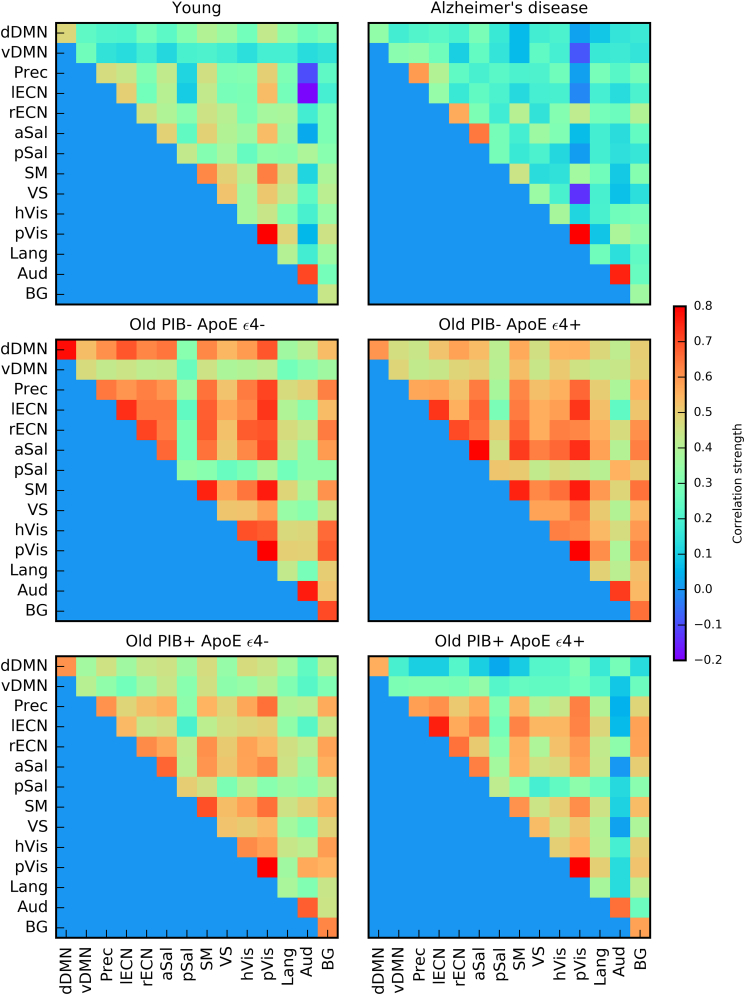
Intrinsic connectivity networks metabolic correlation strengths for each subgroup. Metabolic correlation strengths shown within- (diagonal) and between- (off diagonal) intrinsic connectivity networks. dDMN = dorsal Default Mode Network, vDMN = ventral Default Mode Network, Prec = Precuneus, lECN = left Executive Control Network, rECN = right Executive Control Network, aSal = anterior Salience, pSal = posterior Salience, SM = sensorimotor, VS = Visuospatial, hVis = high Visual, pVis = primary Visual, Lang = Language, Aud = Auditory, BG = Basal Ganglia.

For each group we examined the relationships within- (diagonal) and between- (off diagonal) ICNs (Fig. 6). Unlike the previously presented graphical whole brain approach, this approach was designed to specifically test the expectation that the dependency of metabolic rate between regions should be greater for regions within the same ICN compared with regions outside of the ICN.

Examination of mean correlation within- and between- ICNs revealed clear qualitative distinction between the metabolic brain networks of young and Alzheimer's disease groups; high correlations for the Alzheimer's disease group were largely restricted to within-ICN associations, whereas the young group exhibited high correlations within most ICNs as well as a rich pattern of high and low correlations between ICNs. Both of the older PIB+ subgroups also exhibited high correlations within most ICNs and a diverse pattern of correlations between ICNs, which generally showed more between-ICN correlations than seen in the young or Alzheimer's disease patients and fewer between-ICN correlations than the PIB- subgroups. The PIB+ ApoE ε4+ between-ICN metabolic correlations were weaker than seen in the PIB+ ApoE ε4- group. The Old PIB- subgroups exhibited a homogeneous pattern of widespread high correlations both within and between most ICNs. Interestingly, some of the most striking differences between the older subgroups were lower between-ICN metabolic correlation strengths between the dorsal DMN and the other ICNS in the older PIB+ subgroups compared with the older PIB- subgroups.

## 4 Discussion

We examined metabolic brain networks of young adults, patients with Alzheimer's disease, and four subgroups of cognitively normal older adults based on the presence or absence of two Alzheimer's risk factors: Aβ deposition and the ApoE ε4 allele. Cognitively normal older adults exhibited widespread high metabolic correlation strength compared to young and Alzheimer's disease subjects. The extent of elevated metabolic correlation was reduced in the subgroup with both Aβ and ApoE ε4 genotype (Fig. 2) and in PIB+ older subgroups generally (Fig. 1). By comparison, young adults and patients with Alzheimer's disease both had lower mean metabolic correlation strength than cognitively normal older adults, with the metabolic correlation strength of Alzheimer's patients being somewhat lower than that of young adults. The pattern of metabolic dependencies across the brain differed between young adults, Alzheimer's patients, older adults with both Aβ and ApoE ε4 genotype, and other older adults.

We also examined metabolic brain network correlations within- and between- canonical resting state ICNs identified using resting-state fMRI by Shirer et al. (2012). These ICNs reflect sub-networks of ROIs that functionally couple at rest and underlie subject-driven cognitive states. Young adults showed relatively high metabolic correlation strength within-ICNs and a rich pattern of varied metabolic correlation strengths between-ICNs. In contrast, Alzheimer's patients showed a dramatic reduction in correlation strength between ICNs. PIB- older adults showed homogeneous high metabolic correlation strength both within- and betweeen-ICNs. PIB+ older adults appeared to be intermediary to Alzheimer's patients and PIB- older adults, exhibiting similar within- and between-ICN correlation patterns to the young subjects. These findings identified previously undescribed alterations in metabolic networks in aging, Alzheimer's disease, and those at the highest Alzheimer's disease risk.

**Fig. 7:**
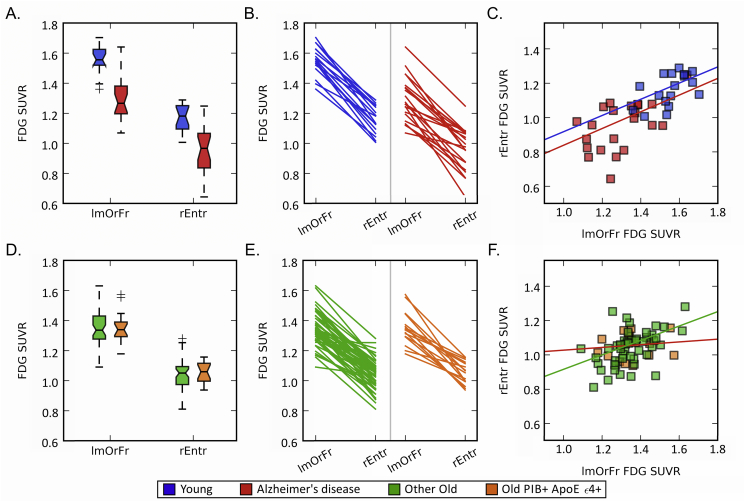
A closer look at univariate versus bivariate relationships.* (A) Boxplots of the distribution of FDG SUVR values for young adults and patients with Alzheimer's disease in two ROIs (lmOrFr = left medial orbitofrontal, rEntr = right entorhinal) reveals univariate differences in FDG SUVR between groups (Young > Alzheimer's disease) and between ROIs (left medial orbitofrontal > right entorhinal). (B) Difference in FDG SUVR values between ROIs for individual participants. Each line segment represents one young adult (blue) or patient with Alzheimer's disease (red). This demonstrates a similar bivariate metabolic relationship between the two ROIs across participants. (C) Scatter plot of the relationship between FDG SUVR values for two ROIs. Each point represents one participant. A regression line shows the relationship of FDG SUVR between the ROIs for each group. The linear relationship of FDG SUVR between the ROIs suggests that metabolism from one ROI predicts metabolism in the other ROI across all of the subjects in the analysis; there is a linear dependence of metabolism between these ROIs across subjects, and both young adults and patients with Alzheimer's disease demonstrate this similarly. Young R^2^ = 0.54, Alzheimer's disease R^2^ = 0.55 (D) Boxplots of the distribution of FDG SUVR values for Other Old and Old PIB+ ApoE ε4+ in two ROIs. This reveals no univariate differences in FDG SUVR for either ROI between groups. (E) Difference in FDG SUVR values between ROIs. Each line segment represents one older adult. This demonstrates group differences in the bivariate metabolic relationship between the two ROIs. (F) Scatter plot of the relationship between FDG SUVR values for two ROIs. Each point represents one participant. Regression line shows the relationship of FDG SUVR between the ROIs for each group. The linear relationships differ between groups; metabolism from one ROI predicts metabolism from the other ROI for the Other Old group, but not for the PIB+ ApoE ε4+ group since that group does not demonstrate a consistent dependence in metabolism between the ROIs. Other Old R^2^ = 0.67, Old PIB+ ApoE ε4+ R^2^ = 0.13. *This example was specifically selected to explain how univariate and bivariate results can differ within the same data, and is not necessarily representative of the dataset as a whole.(For interpretation of the references to color in this figure legend, the reader is referred to the web version of this article.)

Metabolic brain networks, which reflect the co-variation in metabolism across individuals, should be interpreted differently than previously reported univariate analyses of metabolism and other analyses of functional brain networks. For example, while Alzheimer's patients may be hypometabolic in two ROIs compared to young adults (Fig. 7A), the relative metabolism in one ROI compared to the other may be preserved (Fig. 7B and C) - demonstrating the presence of a univariate group difference, but not a bivariate difference between groups in these ROIs. Alternately, while the metabolic rate may be indistinguishable across subgroups of older adults in two ROIs (Fig. 7D), the groups may demonstrate different patterns of linear dependence between the two ROIs (Fig. 7E and F) - demonstrating the presence of a bivariate group difference, but not a univariate difference between groups. Thus, significant univariate results do not imply significant bivariate results, and vice versa. Moreover, unlike functional brain networks, which utilize fluctuations in brain activity over time, metabolic brain networks utilize fluctuations in metabolism across individuals to infer dependence of metabolism in ROIs (Fig. 7C and F). High metabolic correlations are consistent with low individual variability in the relative metabolism between brain regions (i.e. “metabolic homogeneity” across individuals), such that metabolism in one region can be used to infer metabolism in another region due to a consistent linear relationship in relative metabolism across individuals. Low metabolic correlations are consistent with high individual variability in the dependency of metabolism between brain regions (i.e. “metabolic heterogeneity” across individuals), such that metabolism in one region cannot be used to infer metabolism in another region due a lack of a consistent linear relationship across individuals. These separate ways of exploring group differences provide distinct insights into the underlying processes of aging and Alzheimer's disease in the brain.

Metabolic brain networks share commonalities with cortical thickness networks, both of which examine covariance between brain regions across subjects. In such networks positive correlations indicate that both ROIs are either increasing or decreasing consistently across subjects, negative correlations indicate that one ROI is increasing and the other is decreasing consistently across subjects, and near-zero correlations indicate inconsistency in the relative rates of the ROIs across subjects. Decreased structural covariance within the DMN has been reported in older adults and patients with MCI and Alzheimer's disease (Spreng and Turner, 2013, Montembeault et al., 2016), however other brain networks were also disrupted and reflected an inverted-U pattern consistent with maximal segregation of functional networks in young adults followed by dedifferentiation in old age (DuPre and Spreng, 2017). Disruption is more exaggerated in patients with Alzheimer's disease (Montembeault et al., 2016) and in ApoE ε4 carriers (Spreng and Turner, 2013). Patients with Alzheimer's disease further exhibited increased local interregional correlations and disrupted long distance correlations (Yao et al., 2010), and altered graph theoretic properties (He et al., 2008, Yao et al., 2010). Our findings in metabolic brain networks of Alzheimer's patients mirror those reported in cortical thickness networks, however the widespread elevation of correlation strength, as we found in metabolic brain networks of older adults, was a divergent finding from previous reports of reductions in correlation strength for cortical thickness networks of older adults. Thus, metabolism may undergo a more homogenous pattern of change in older adults, compared to a more heterogeneous pattern of atrophy across individuals. Importantly, studies of cortical thickness networks in older adults have not accounted for the effects of Aβ. Exploration of preclinical Alzheimer's pathology on cortical thickness networks in old age would further clarify the relationship between the processes of atrophy and metabolic change, which have shown evidence of divergence in univariate studies (Ibáñez et al., 1998, Chételat et al., 2008, La Joie et al., 2012, Grothe et al., 2016, Kljajevic et al., 2014) and metabolic brain networks (Di et al., 2012).

Widespread elevations in correlation strength of metabolic brain networks observed in cognitively normal older adults suggest a novel phenomenon in aging - metabolic homogeneity. Previous work posited that weaker metabolic correlation strength in Alzheimer's disease reflects “metabolic heterogeneity” due to variability in compensatory and/or degenerative process that lead to inter-individual variability in metabolism (Carbonell et al., 2014, Sanabria-Diaz et al., 2013). We found the opposite effect - very strong metabolic correlation strength - in cognitively normal older adults, which would be consistent with “metabolic homogeneity”. Interestingly, this homogeneity must be occurring despite the presence of age-related compensatory and degenerative processes. As white matter integrity typically decreases in old age, the pervasive metabolic “hyper-connectivity” observed in the present study does not reflect increased structural connectivity between brain regions. Rather, this phenomenon may reflect dedifferentiation that occurs with age-related loss of aerobic glycolysis (Goyal et al., 2017), functional connectivity (Geerligs et al., 2015), white matter integrity (Andrews-Hanna et al., 2007, Saenger et al., 2017), and BOLD variability (Garrett et al., 2011) that negatively affect dynamic exploration of functional brain states (McIntosh et al., 2010, Deco et al., 2011). Our finding that reduction of correlations was related to further anatomical distance in two of the normal older groups (Section 3.4) may be consistent with white matter alterations leading to metabolic homogeneity. Further work is needed to explore age-related metabolic homogeneity, including its potential relationship with aerobic glycolysis, functional connectivity, white matter alterations, and other measures of brain structure and function, as well as the effects of deviation from this old age-related metabolic correlation profile on brain function and degeneration.

Metabolic homogeneity may be a form of “dedifferentiation”, an age-related process previously posited in light of reductions in hemispheric asymmetry (Dolcos et al., 2002), loss of functional specialization (Park et al., 2004), and reduced task-related deactivation (Prakash et al., 2012). Age-related dedifferentiation was further confirmed by our ICN analysis, which revealed widespread high between-ICN metabolic correlation strength in cognitively normal older adults, indicative of desegregation. This was true across all subgroups of older adults, regardless of Aβ and ApoE status, although desegregation was greater in older subgroups without Aβ compared to those with Aβ which may be indicative of divergent process of aging and Alzheimer's disease. Segregation of the brain into functionally specialized subnetworks is a key organizational feature of structural and functional brain networks that supports differentiation of brain function (Chen et al., 2008, He et al., 2009). Studies in other modalities have also demonstrated age-related desegregation of brain networks (Chen et al., 2011, Geerligs et al., 2015), as well as step-wise decreases in segregation with Alzheimer's disease severity (Brier et al., 2014). Overall, widespread elevated metabolic correlation was consistent with a profound loss of independence in metabolism across brain systems in normal aging, leading to dedifferentiation and desegregation of metabolism.

Individuals possessing both Aβ and the ApoE ε4 genotype appeared to be on an altered metabolic trajectory compared to other cognitively normal older adults without both risk factors. Because the network correlation pattern of this group was intermediate between young and Alzheimer's disease patients, and quite different from their normal old-aged peers, the altered trajectory could represent either preservation of youth-like function or the start of decline towards Alzheimer's disease. While the latter seems more likely, increased neural activity that might be associated with persistence of youth-like metabolic function has been proposed as an underlying mechanism linking ApoE genotype, Aβ, and aging (Jagust and Mormino, 2011, Oh et al., 2016). However, the relatively low metabolic correlation strength in the entorhinal cortex and temporal lobe - regions that exhibit marked neurodegeneration in Alzheimer's disease (Du et al., 2001) as well as neurodevelopmental differences in early life (Shaw et al., 2007) - bore intriguing similarity to the Alzheimer's group (Fig. 3, Fig. 4). Moreover, prior work demonstrating an interaction of Aβ and ApoE ε4 genotype in healthy older adults reported lower cognitive performance (Kantarci et al., 2012) and faster rates of cognitive decline (Mormino et al., 2014) only in subjects with both risk factors. Various mechanisms may make ApoE ε4 carriers more vulnerable to the toxic effects of Aβ, including alterations in tau phosphorylation, neuroinflammation, mitochondrial function, synaptic function, and/or neurodevelopmental differences in cortical thickness and connectivity (Wolf et al., 2013, Brown and Jessup, 2009). However, another possibility is that individuals with both risk factors were further along the Alzheimer's disease continuum, given the younger age of onset of Alzheimer's disease in patients with ApoE ε4 genotype (Corder et al., 1993).

Due to the plurality and diversity of age-related processes, the study of aging is rife with confounding variables and ultimately it is beyond the scope of any single study to address all of these challenges. The major limiting factor of the present study was the small number of subjects. We attempted to control for spurious results by confirming consistency using permutation testing, which helped protect against individual subject or a subset of subjects having undue influence on the results, and conducted some analyses on a larger group (i.e. Other Old, N = 48) composed of all older adults except those in the PIB+ ApoE ε4+ group (which remained lower powered at N = 16). The limited number of subjects precluded the use of partial correlations to control for additional variables. However, we were able to match subgroups for sex, years of education, and, when appropriate, age. While brain atrophy and partial volume effects consequent to the relatively low resolution of PET will increase regional metabolic covariance, this did not seem likely to explain the pattern of results (see Section 3.4). Moreover, atrophy was unlikely to explain opposite effects on metabolic correlation strength: elevated metabolic correlation strength in cognitively normal older adults, but reduced metabolic correlation strength in Alzheimer's patients. We conducted the analysis using pons-normalized fully-connected weighted graphs for each group, and thus our results should not be directly compared with studies using binary graphs or partially-connected (i.e. thresholded) graphs, whole-brain-normalized graphs, as well as graphs generated from other neuroimaging modalities. However, our results suggested that there may be significant differences in the appropriate “connection density” (i.e. number of edges in a graph) between groups, providing a strong case against the use of binary graphs or graphs thresholded based on connection density when examining age- and disease-related differences in metabolic brain networks (and possibly other neuroimaging modalities as well). We recognize that glucose metabolism is a complex phenomenon reflecting multiple metabolic processes (Zimmer et al., 2017). Nevertheless, it is clearly related to synapse structure and function as well as measures of brain function and connectivity using multiple modalities (Riedl et al., 2014, Tomasi et al., 2013, Rocher et al., 2003, Goyal et al., 2014).

In conclusion, metabolic brain networks revealed distinct effects of aging and Alzheimer's disease risk on metabolic processes in cognitively normal older adults. We identified a previously undescribed process of widespread elevated metabolic correlation in aging, which disrupted the segregation of ICNs across the brain. Moreover, we demonstrated that the metabolic brain network of normal older adults with both Aβ and ApoE ε4 genotype differed substantially from that of their normal old-aged peers without both risk factors, possessing a pattern of metabolic correlations that is more similar to that of young adults and Alzheimer's disease patients. Analysis based on ICNs further distinguished PIB+ from PIB- older adults, showing greater dedifferentiation in PIB- subgroups and a profile more similar to Alzheimer's patients in PIB+ subgroups. The effect of dual Alzheimer's risk factors appeared to be much more prominent when examining metabolic brain networks than the weak and inconsistent effects identified using other approaches, suggesting that the alterations captured by metabolic brain networks may be especially important for understanding cognitive decline and progression to Alzheimer's disease. While the clinical applications of these results are unclear, the findings of alterations in metabolic networks that differ significantly in aging and those at high risk of Alzheimer's disease may motivate the exploration of these effects in the further search for biomarkers and mechanisms important in the earliest stages of Alzheimer's disease.

## Appendix A Supplementary data

Table S1 Numerical labels and ICN assignments for all regions from the Freesurfer Desikan-Killiany atlas. Table S1
