# Supplementary material for: Metabolic brain networks in aging and preclinical Alzheimer's disease
Source: Neuroimage Clin. 2017 Dec 28;17:987–99. doi: 10.1016/j.nicl.2017.12.037 (PMC5842784; doi:10.1016/j.nicl.2017.12.037)
Supplement: Table S1 — Numerical labels and ICN assignments for all regions from the Freesurfer Desikan-Killiany atlas. [file mmc1.docx]

| **#** | **Freesurfer ROI** | **Lobe** | **#** | **Freesurfer ROI** | **ICN** |
| --- | --- | --- | --- | --- | --- |
| 1 | ctx-lh-caudalanteriorcingulate | Cingulate | 41 | ctx-rh-caudalanteriorcingulate | Cingulate |
| 2 | ctx-lh-isthmuscingulate | Cingulate | 42 | ctx-rh-isthmuscingulate | Cingulate |
| 3 | ctx-lh-posteriorcingulate | Cingulate | 43 | ctx-rh-posteriorcingulate | Cingulate |
| 4 | ctx-lh-rostralanteriorcingulate | Frontal | 44 | ctx-rh-rostralanteriorcingulate | Frontal |
| 5 | ctx-lh-caudalmiddlefrontal | Frontal | 45 | ctx-rh-caudalmiddlefrontal | Frontal |
| 6 | ctx-lh-frontalpole | Frontal | 46 | ctx-rh-frontalpole | Frontal |
| 7 | ctx-lh-lateralorbitofrontal | Frontal | 47 | ctx-rh-lateralorbitofrontal | Frontal |
| 8 | ctx-lh-medialorbitofrontal | Frontal | 48 | ctx-rh-medialorbitofrontal | Frontal |
| 9 | ctx-lh-paracentral | Frontal | 49 | ctx-rh-paracentral | Frontal |
| 10 | ctx-lh-parsopercularis | Frontal | 50 | ctx-rh-parsopercularis | Frontal |
| 11 | ctx-lh-parsorbitalis | Frontal | 51 | ctx-rh-parsorbitalis | Frontal |
| 12 | ctx-lh-parstriangularis | Frontal | 52 | ctx-rh-parstriangularis | Frontal |
| 13 | ctx-lh-precentral | Frontal | 53 | ctx-rh-precentral | Frontal |
| 14 | ctx-lh-rostralmiddlefrontal | Frontal | 54 | ctx-rh-rostralmiddlefrontal | Frontal |
| 15 | ctx-lh-superiorfrontal | Frontal | 55 | ctx-rh-superiorfrontal | Frontal |
| 16 | ctx-lh-insula | Insula | 56 | ctx-rh-insula | Insula |
| 17 | ctx-lh-cuneus | Occipital | 57 | ctx-rh-cuneus | Occipital |
| 18 | ctx-lh-lateraloccipital | Occipital | 58 | ctx-rh-lateraloccipital | Occipital |
| 19 | ctx-lh-lingual | Occipital | 59 | ctx-rh-lingual | Occipital |
| 20 | ctx-lh-pericalcarine | Occipital | 60 | ctx-rh-pericalcarine | Occipital |
| 21 | ctx-lh-inferiorparietal | Parietal | 61 | ctx-rh-inferiorparietal | Parietal |
| 22 | ctx-lh-postcentral | Parietal | 62 | ctx-rh-postcentral | Parietal |
| 23 | ctx-lh-precuneus | Parietal | 63 | ctx-rh-precuneus | Parietal |
| 24 | ctx-lh-superiorparietal | Parietal | 64 | ctx-rh-superiorparietal | Parietal |
| 25 | ctx-lh-supramarginal | Parietal | 65 | ctx-rh-supramarginal | Parietal |
| 26 | Left-Amygdala | Subcortical | 66 | Right-Amygdala | Subcortical |
| 27 | Left-Caudate | Subcortical | 67 | Right-Caudate | Subcortical |
| 28 | Left-Cerebellum-Cortex | Subcortical | 68 | Right-Cerebellum-Cortex | Subcortical |
| 29 | Left-Hippocampus | Subcortical | 69 | Right-Hippocampus | Subcortical |
| 30 | Left-Pallidum | Subcortical | 70 | Right-Pallidum | Subcortical |
| 31 | Left-Putamen | Subcortical | 71 | Right-Putamen | Subcortical |
| 32 | Left-Thalamus-Proper | Subcortical | 72 | Right-Thalamus-Proper | Subcortical |
| 33 | ctx-lh-entorhinal | Temporal | 73 | ctx-rh-entorhinal | Temporal |
| 34 | ctx-lh-fusiform | Temporal | 74 | ctx-rh-fusiform | Temporal |
| 35 | ctx-lh-inferiortemporal | Temporal | 75 | ctx-rh-inferiortemporal | Temporal |
| 36 | ctx-lh-middletemporal | Temporal | 76 | ctx-rh-middletemporal | Temporal |
| 37 | ctx-lh-parahippocampal | Temporal | 77 | ctx-rh-parahippocampal | Temporal |
| 38 | ctx-lh-superiortemporal | Temporal | 78 | ctx-rh-superiortemporal | Temporal |
| 39 | ctx-lh-temporalpole | Temporal | 79 | ctx-rh-temporalpole | Temporal |
| 40 | ctx-lh-transversetemporal | Temporal | 80 | ctx-rh-transversetemporal | Temporal |

Table S1: **Numerical labels and ICN assignments for all regions from the Freesurfer Desikan-Killiany atlas.**
